# Case report: a giant cell-rich gnathic bone lesion in a child with pycnodysostosis

**DOI:** 10.3389/froh.2023.1188443

**Published:** 2023-05-23

**Authors:** C. Spencer, A. Makka, S. Singh, J. McGuire, N. Washaya, G. Hein, M. Zampoli, K. Fieggen

**Affiliations:** ^1^ Division of Human Genetics, University of Cape Town, Cape Town, South Africa; ^2^ Division of Human Genetics, Department of Medicine, Groote Schuur Hospital, Cape Town, South Africa; ^3^ Department of Maxillo-Facial and Oral Surgery, University of the Western Cape, Cape Town, South Africa; ^4^ Division of Anatomical Pathology, University of Cape Town and National Health Laboratory Services, Cape Town, South Africa; ^5^ Division of Otolaryngology, University of Cape Town, Cape Town, South Africa; ^6^ Division of Paediatric Pulmonology, Department of Paediatrics and Child Health, University of Cape Town, Cape Town, South Africa; ^7^ Department of Paediatrics, National University of Science and Technology, Harare, Zimbabwe

**Keywords:** pycnodysostosis, giant cell-rich, obstructive sleep apnea, gnathic bones, case report

## Abstract

Pycnodysostosis is a skeletal dysplasia characterized by short stature, generalized osteosclerosis, acro-osteolysis, and recognizable facial features. Oral manifestations are commonly described and include a high-arched palate with dental crowding and malocclusion, hypoplastic enamel, and retained deciduous teeth with impacted permanent teeth, and there is an increased risk of developing osteomyelitis of the jaw. We report here the history of a 9-year-old male with the typical facial and skeletal phenotype of pycnodysostosis but novel oral features. He presented with bilateral progressive facial swelling, which caused functional impairment with chewing and contributed to his severe obstructive sleep apnea (OSA). The severity of his OSA required surgical intervention, and the lesions were resected. Extensive bone remodeling and replacement by fibrous tissue were noted on submucosal dissection, and bilateral subtotal maxillectomies were required. The histopathology of the biopsied lesion was consistent with a giant cell-rich lesion. Genetic testing identified a pathogenic homozygous variant in the *CTSK* gene, c.953G > A, p. (Cys318Tyr). The proband had a good postsurgical response with sustained improvement in his sleep OSA. We present here the history and clinical characteristics of a patient with typical features of pycnodysostosis and an unusual presentation and histopathology of gnathic bone lesions. This report adds to the body of literature on this rare condition and also highlights the finding of giant cell-rich lesions of the gnathic bones. Giant cell-rich lesions in pycnodysostosis have previously been reported in two cases in the literature. While there is not enough evidence to support a certain association with pycnodysostosis, it is prudent to consider regular oral dental reviews in affected individuals to identify pathology early and avoid such life-threatening complications.

## Introduction

Pycnodysostosis (OMIM #265800) is a skeletal dysplasia classified as an osteopetrosis condition (1). It is characterized by short stature, generalized osteosclerosis, acro-osteolysis, and recognizable facial features (frontal bossing, proptosis, convex nasal bridge, micrognathia, and an obtuse mandibular angle) (2). Additional features include delayed closure of fontanelles, dysplastic acromial clavicular ends, and scoliosis.

Oral manifestations are commonly described and include a high-arched palate with dental crowding and malocclusion, hypoplastic enamel, and retained deciduous teeth with impacted permanent teeth, and there is an increased risk of developing osteomyelitis of the jaw (3, 4). Other complications, such as a high risk of fractures and hearing loss, can also occur. Respiratory manifestations frequently reported include stridor, laryngomalacia, and OSA, the latter is often severe enough to require noninvasive ventilatory support (2).

Pycnodysostosis is a rare autosomal recessive condition with a prevalence of 1–3/1,000,000 people (2). It is caused by pathogenic variants in the *CTSK* gene. This gene encodes cathepsin K, which produces a lysosomal cysteine protease that is highly expressed in osteoclasts and plays a role during bone resorption and remodeling. Loss of function variants are known to be causative in pycnodysostosis. The osteosclerotic phenotype is explained by the lack of lysosomal cysteine protease, while osteolysis is thought to be due to a site-specific variation in the enzyme action causing increased bone resorption (5).

Giant cell-rich lesions are a heterogeneous group of non-neoplastic and neoplastic tumors that usually develop from bone and are recognized by the presence of a number of reactive osteoclast-type multinucleate giant cells (6). The entities comprising this group show marked overlap in histological features, and thus, there is a need to consider clinical and molecular aspects along with the histopathological findings to make a clear diagnosis.

We present a proband with pycnodysostosis, undiagnosed prior to presentation, complicated by OSA and bilateral infiltrating, giant cell-rich lesions of the gnathic bones. To the best of our knowledge, this is only the third documentation of a giant cell-rich lesion in this condition.

## Case description

### Clinical presentation

The proband, a 9-year-old boy of Zimbabwean ancestry, presented to Red Cross War Memorial Children's hospital (RCWMCH) with bilateral, progressively enlarging cheek masses, airway obstruction, and difficulty chewing food.

He was born to his non-consanguineous parents at term after an uneventful pregnancy with normal birth growth parameters; his postnatal course was uncomplicated, with no early failure to thrive. At 1 year of age, he presented with upper airway obstruction initially conservatively managed, but he later underwent an adenotonsillectomy at 3 years. Despite this, his upper airway obstructive symptoms remained a concern. At 8 years of age, he was noted to have swelling of both cheeks, which progressively worsened.

**Figure 1:**
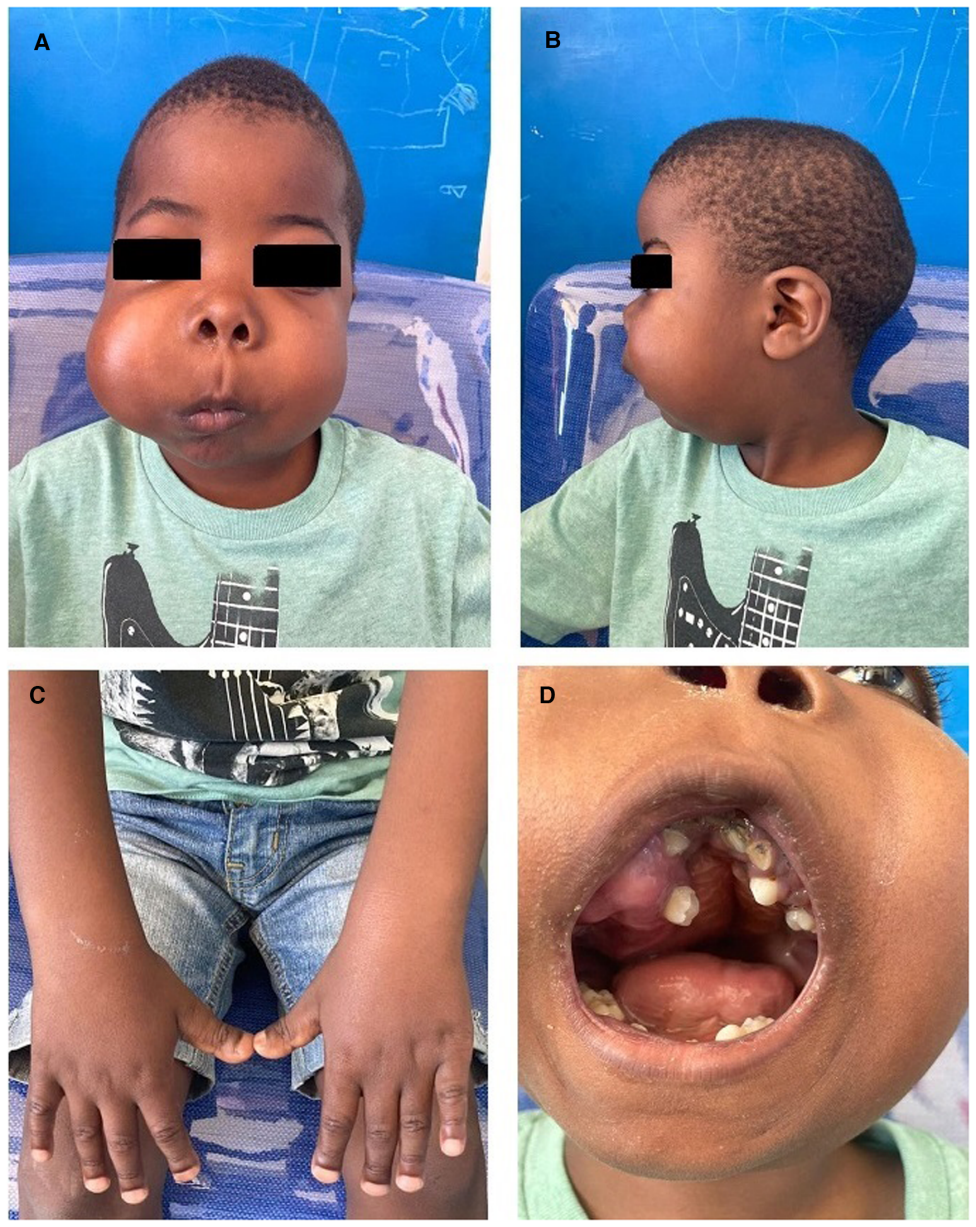
(**A**) Frontal photograph revealing bilateral swelling, right more severe than left, with bitemporal narrowing and a long philtrum. (**B**) Lateral photograph showing retrognathia. (**C**) Marked brachydactyly with broad distal phalanges. (**D**) Intraoral mass with dental crowding, malocclusion, and a high-grooved palate.

At a presentation at 9 years, the proband's growth parameters showed height and weight below the 3rd centile and a head circumference on the 25th centile. His facial features were striking for asymmetrically swollen cheeks. Additional clinical findings included bifrontal narrowing with frontal prominence, proptosis, a flattened nasal bridge with a convex nasal profile, a long philtrum, and retrognathia (Figures 1A,B). He had brachydactyly with broad short distal phalanges (Figure 1C). He also demonstrated small joint laxity with genu varus and pes planus. His anterior fontanelle was still patent. There were no cognitive delays.

The right-sided facial mass was approximately 3 cm × 4 cm, with multiple smaller mobile lesions that were not tender. The left-sided lesion was smaller, measuring 2 cm × 1 cm, also with smaller mobile lesions, some of which were tender. Oral examination showed prominent dental decay, over-retained primary teeth, impacted permanent teeth with displacement and overcrowding, and intraoral extension of the mass lesions (Figure 1D). The palate was intact. A formal otorhinolaryngology assessment confirmed bilateral nasal stenosis due to external compression and remodeling from the lesions.

**Figure 2:**
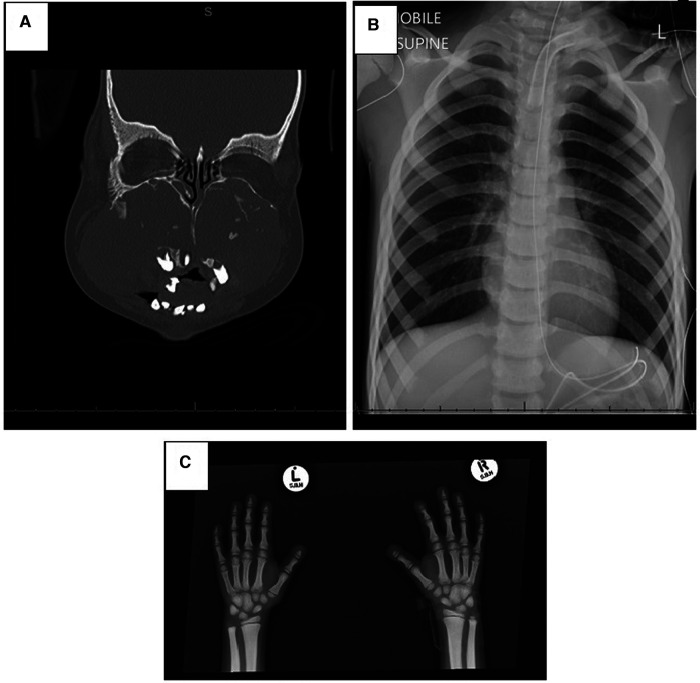
Radiological investigations. (**A**) CT scan of the face showing expansile, mixed-density masses involving the maxilla and mandible bilaterally. The maxillary lesions are large, resulting in remodeling of the maxillary bones causing obliteration of the maxillary sinuses, nasal cavity remodeling and obstruction, atrophy of the hard palate, and displacing the teeth. (**B**) Chest x-ray with generalized sclerosis. (**C**) Osteolysis of distal phalanges and generalized sclerosis.

Several special investigations were conducted; skeletal x-rays showed generalized sclerosis with acro-osteolysis, and a CT scan of his head revealed expansile mixed-density masses involving the maxillae and mandible bilaterally (Figure 2). The mandibular lesions had shown spontaneous regression over a 6-year period compared to earlier images. The proband's sleep study on admission showed severe OSA associated with periods of hypoventilation. Blood tests revealed normal renal function, calcium, phosphate, and parathyroid hormone and did not support an underlying infective or inflammatory cause.

### Management

The multifactorial OSA was severe, and a decision was made to intervene surgically and resect the lesions. During surgery, the otorhinolaryngology and maxilla-facial team secured the airway with a tracheostomy and proceeded to resect the lesions via an oral approach and vestibular incision. Extensive bone remodeling and replacement by fibrous tissue were noted on submucosal dissection, and bilateral subtotal maxillectomies were required. The mandibular lesion was not removed, as further spontaneous regression was expected. Two weeks later, the patient returned to the theatre for impressions and obturator construction, which was subsequently inserted when the tracheostomy was decannulated. The proband showed a good response with marked improvement in his OSA postsurgery.

**Figure 3:**
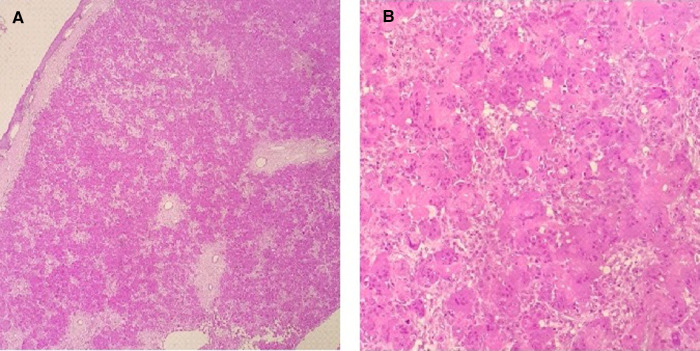
(**A**) Low-power view showing mucosal stratified squamous epithelium with an unencapsulated lesion in the subepithelial stroma. (**B**) Lesion is predominantly composed of aggregates of multinucleated osteoclast-type giant cells with admixed mononuclear spindle cells.

The histopathology of the biopsied lesion showed aggregates of multinucleated osteoclast-type giant cells with admixed mononuclear spindle cells and intervening fibrous bands (Figure 3). Mitotic activity and apoptosis were observed. The lesion extended into adjacent skeletal muscle and bone. The results were compatible with a giant cell-rich lesion. No features of osteomyelitis were found clinically or on histopathology, and no calcifications were visible.

### Genetic testing

Genetic testing was undertaken in the proband with a commercially available next-generation sequencing osteogenesis and bone fragility panel (Test code: 04732, Invitae, USA). The result showed a pathogenic homozygous variant of the *CTSK* gene, NM_000396.3:c.953G > A, p. (Cys318Tyr). Notably, no pathogenic or uncertain variants were found in *SH3PB2*, the gene responsible for cherubism initially suspected in the proband. The diagnosis of pycnodysostosis was confirmed, and the family and proband counseled accordingly. Genetic testing of the tumor tissue was not undertaken.

## Discussion

We present the history and clinical course of a Zimbabwean child with pycnodysostosis who presented with bilateral giant cell-rich lesions of his gnathic bones. While the clinical features were typical of pycnodysostosis, the oral manifestations present in this child have not been as well delineated. This highlights the need for regular and careful oral and dental reviews in this rare condition.

The proband presented with progressive facial swelling found to be giant cell-rich lesions of his mandible and maxillae, contributing to his severe airway obstruction. Examples of giant cell-rich lesions seen in the head and neck area include giant cell granuloma, cherubism, giant cell tumor of the bone, aneurysmal bone cyst, tenosynovial giant cell tumor, phosphaturic mesenchymal tumor, and brown tumor of hyperparathyroidism (6). The presence of a central giant cell-rich lesion should prompt an endocrine work-up, which in the case of this patient was normal. Cherubism is caused, in most cases, by variations in *S3HBP2.* In a few cases, despite a clinical diagnosis, a molecular cause is not found, suggesting possible genetic heterogeneity (7). Although initially suspected to have cherubism, the absence of a variant of *S3HBP2*, in this case, makes that highly unlikely.

The oral manifestations in pycnodysostosis previously described include dental crowding, a grooved and high-arched palate, malocclusion of teeth with hypoplastic enamel, hypercementosis, and often retained deciduous teeth with impacted permanent teeth. One of the important complications facing patients with pycnodysostosis is osteomyelitis (4). The lesions observed in this proband were not in keeping with osteomyelitis. Giant cell-rich tumors are only reported in two other patients with pycnodysostosis in the literature. One publication reports on a 10-year-old female who presented with increased swelling of the jaw following tooth extraction several months earlier; an incisional biopsy diagnosed a central giant cell lesion. Years later, this patient developed new lesions confirmed to be benign fibro-osseous lesions (8). A second publication describes an adult woman who presented with an incidental finding of a giant cell tumor of her occipital bone (9). Only the second publication mentions the causative variant in intron 2 of *CTSK* (c.120 + 1G > T), which does not share a common protein domain with the current patient's variant in exon 8. In addition, only the first patient reported a traumatic event preceding the development of the giant cell-rich lesion. With fewer than 500 cases of pycnodysostosis reported (2), there is insufficient evidence to discern whether these lesions are incidental or have a rare association with the condition. Giant cell-rich lesions of the bone are however uncommon tumors seen in children and are most often described in older individuals (6).

There are more than 35 *CTSK* variants associated with pycnodysostosis (2); the proband in this report has a known pathogenic variant, c.953G > A (p.Cys318Tyr). This is a well-described pathogenic variant described in 10 affected individuals. In eight of these cases, the variant was found in a homozygous state; descriptions have included seven patients from Brazil, two siblings from Comoros, and one from Sao Tome (2, 5, 10, 11). Girbal et al. suggested that a more severe phenotype with life-threatening OSA and anemia may be associated with this variant; however, this has not been confirmed in other cases, and larger studies have not shown clear genotype–phenotype correlations as yet (2, 11). The patient described here did not have anemia but severe OSA secondary to large gnathic bone lesions.

To summarize, we present here the history and clinical characteristics of a patient with typical features of pycnodysostosis but an unusual presentation and histopathology of gnathic bone lesions. This report adds to the literature on this rare condition and also highlights the finding of giant cell-rich lesions of the gnathic bones. While there is not enough evidence to support a certain association with pycnodysostosis, it is prudent to consider regular oral dental reviews in affected individuals to identify pathology early and avoid such life-threatening complications.

## Data Availability

The raw data supporting the conclusions of this article will be made available by the authors without undue reservation.
